# Brain correlates of motor complexity during observed and executed actions

**DOI:** 10.1038/s41598-020-67327-5

**Published:** 2020-07-03

**Authors:** Xinge Li, Manon A. Krol, Sahar Jahani, David A. Boas, Helen Tager-Flusberg, Meryem A. Yücel

**Affiliations:** 1 School of Psychology, South China Normal University, Guangzhou, China; 2 Neurophotonics Center, Department of Biomedical Engineering, Boston University, Boston, MA USA; 3 Center for Autism Research Excellence, Department of Psychological and Brain Sciences, Boston University, Boston, MA USA; 4 Donders Institute, Radboud University Medical Center, Nijmegen, The Netherlands; 5 MGH/HST Athinoula A. Martinos Center for Biomedical Imaging, Department of Radiology, Massachusetts General Hospital, Harvard Medical School, Charlestown, MA USA

**Keywords:** Motor cortex, Premotor cortex

## Abstract

Recently, cortical areas with motor properties have attracted attention widely to their involvement in both action generation and perception. Inferior frontal gyrus (IFG), ventral premotor cortex (PMv) and inferior parietal lobule (IPL), presumably consisting of motor-related areas, are of particular interest, given that they respond to motor behaviors both when they are performed and observed. Converging neuroimaging evidence has shown the functional roles of IFG, PMv and IPL in action understanding. Most studies have focused on the effects of modulations in goals and kinematics of observed actions on the brain response, but little research has explored the effects of manipulations in motor complexity. To address this, we used fNIRS to examine the brain activity in the frontal, motor, parietal and occipital regions, aiming to better understand the brain correlates involved in encoding motor complexity. Twenty-one healthy adults executed and observed two hand actions that differed in motor complexity. We found that motor complexity sensitive brain regions were present in the pars opercularis IFG/PMv, primary motor cortex (M1), IPL/supramarginal gyrus and middle occipital gyrus (MOG) during action execution, and in pars opercularis IFG/PMv and M1 during action observation. Our findings suggest that the processing of motor complexity involves not only M1 but also pars opercularis IFG, PMv and IPL, each of which plays a critical role in action perception and execution.

## Introduction

Recent advances in cognitive neuroscience have shown that individuals can access and understand others’ actions and intentions not only through meta-cognitive processes, inferential and propositional reasoning, but also in a direct, pre-cognitive and motor-based way, thus linking motor cognition to social cognition^1,2^. Here, motor cognition refers to the functional roles that motor-related cortical areas play in understanding one’s own and others’ actions. Those motor-related areas include pars opercularis of the inferior frontal gyrus (IFG), ventral premotor cortex (PMv) and the rostral part of inferior parietal lobule (IPL). Not surprisingly, a large number of functional neuroimaging studies have found overlapped activation between action observation and execution in these brain regions^3–11^. These motor-related areas have been reported to show motor properties, while they are not constrained to motor processing given their engagement in multiple cognitive functions, e.g., language and spatial attention^12,13^. Some studies further considered aforementioned brain regions to be part of the Mirror Neuron System (MNS), which is considered to be a neural mechanism that is involved in action understanding^14–18^.

So far, converging neuroimaging studies have unraveled the functional roles that these motor-related areas play in action goal and intention understanding. A series of fMRI studies provided strong evidence that pars opercularis IFG, PMv and IPL responded particularly to goal-directed actions, even when actions with the same motor goal were performed with different effectors^19,20^, when the same action was just heard^5,21^; or with different motor goals^22–25^. Moreover, it has been indicated that some of these motor-related areas subserve the understanding of intentions underlying the actions of others. For example, an fMRI study has shown that encoding of potential action intentions resulted in increased activation in right pars opercularis of IFG compared to mere encoding of kinematic features of actions^26^. Furthermore, superior temporal sulcus (STS) has been shown to play a role in processing higher-order visual inputs of goal-directed actions and projecting those visual inputs to pars opercularis IFG, PMv and IPL^14, 16–18^. Therefore, it has been indicated that STS closely interacts with pars opercularis of IFG, PMv and IPL via visual processing during action perception.

While previous work has focused on the effects of modulations in goals and kinematics of observed actions on the brain response, little research has explored the effects of manipulations in motor complexity. Here, motor complexity refers to the motoric complexity of goal-directed actions. Compared to simple actions, complex actions require more motor preparation and planning to establish a state of readiness to make a specific planned movement. Using fMRI, Molnar-Szakacs and colleagues investigated the brain response during observation of action sequences with varying complexity^27^. They found that action sequences that were motorically more difficult resulted in significantly more activation in posterior IFG, adjacent PMv and IPL. Biagi and colleagues also looked at the brain correlates of motor complexity of the observed action^28^. They found that anterior intraparietal area (AIP) was more active during the observation of complex object-manipulation tasks compared to simple tasks. Both studies provide insight into how one’s own brain is engaged during the observation of other’s actions. However, due to the constrained fMRI environment, neither of these studies included the execution of hand actions by the observer. When investigating motor complexity, it is important to include the actual execution task in addition to the observation task, to ensure that, when performing goal-directed hand actions, these hand actions really differ in terms of motor complexity based on the brain activity in motor and sensorimotor areas. Moreover, given that the functional roles of those motor-related areas have been mainly determined by the overlap of brain activation during action observation and execution, the inclusion of both execution and observation tasks facilitates the interpretation of findings. In this study, we aimed to investigate the brain correlates of observing and executing the same hand action with differing motor complexity using functional Near Infrared Spectroscopy (fNIRS).

Functional near infrared spectroscopy (fNIRS) is an established optical imaging method that uses near-infrared light to noninvasively quantify the hemodynamic responses associated with neural activity, by measuring the concentration changes of oxy-hemoglobin (HbO), deoxy-hemoglobin (HbR) and total hemoglobin (HbT)^29^. The increased blood flow evoked by neural activity in a brain region usually results in an increase in HbO and a decrease in HbR. Previous studies have shown that HbO and HbR responses obtained by fNIRS are temporally and spatially correlated with the Blood Oxygen Level-Dependent signal (BOLD) from fMRI^30–32^. The general advantages of fNIRS are its non-invasiveness, portability and cost-effectiveness^33^. fNIRS is also relatively robust to motion artifacts and allows participants to perform goal-directed hand actions with fairly large and flexible limb movements, and thus it is well suited to study the activation in specific motor-related areas.

In this study, we focused on the brain correlates underlying the overall processing of the motor complexity. Hence, we used fNIRS to examine the brain activity in large-scale cortical areas involved in either motor or visual processing, including pars opercularis IFG and adjacent PMv, primary motor cortex (M1), IPL and supramarginal gyrus, temporo-parietal junction (TPJ), STS and middle occipital gyrus (MOG). Specifically, we kept the goals and kinematics of the actions fairly consistent across conditions while manipulating the amount of motor planning and precision that are involved in the actions. We hypothesized that, as a result of more intense engagement of motor planning and precision, compared to simple actions, increased hemodynamic responses would be recorded from the above motor and motor-related areas not only during the execution of more complex actions but also during the observation of such actions. Moreover, since we controlled the visual inputs during action observation and control conditions, we do not expect to see any contrast in visual areas during observation.

## Materials and methods

### Participants

Twenty-one healthy adults with an average age of 33.5 years (± 15.5) (12 females; 19 right-handed) were enrolled in this study. All participants had normal or corrected-to-normal visual acuity and had no history of neurological or psychiatric disorders. The study was approved by the Institutional Review Board of Massachusetts General Hospital (MGH) and was performed in accordance with relevant guidelines and regulations. All participants gave a written informed consent to take part in the experiment.

### Probe placement and fNIRS system

**Figure 1:**
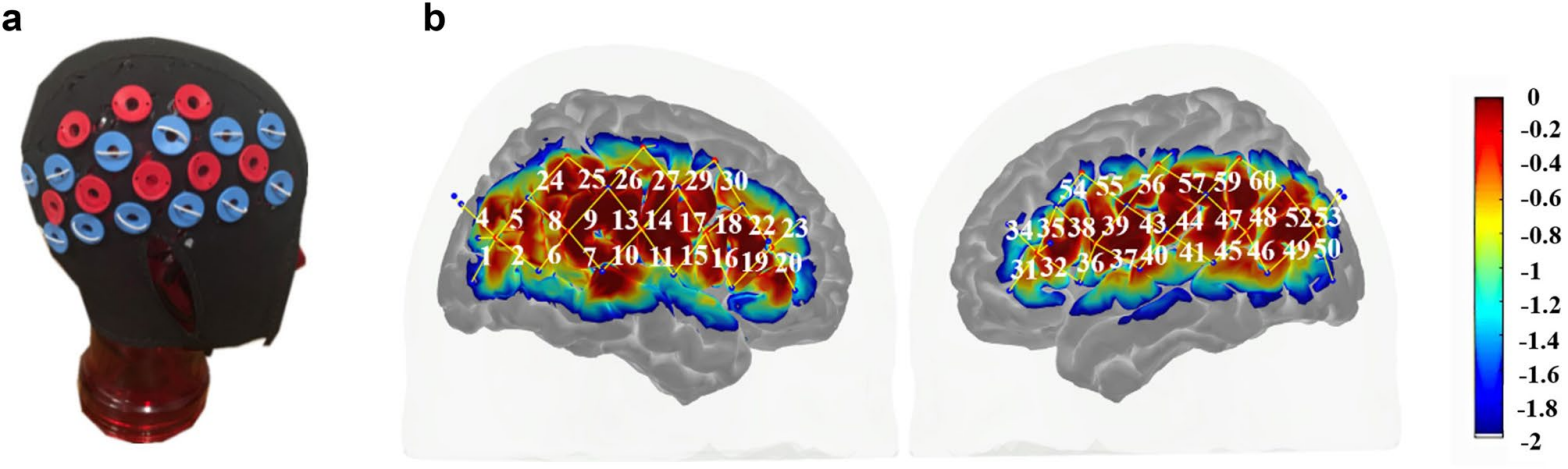
The probe design. (**a**) Cap with sources (red grommets) and detectors (blue grommets), (**b**) sensitivity profile for the probe geometry. The color scale indicates the relative sensitivity in log10 units from − 2 (blue) to 0 (red). The yellow lines and white numeration represent channels and channel numbers respectively. (Short separation-channel numbers are excluded in this figure).

Data were collected by a CW7 NIRS system operating at 690 and 830 nm wavelengths (TechEn Inc. MA, USA) with a 25 Hz sampling frequency. The NIRS probe was designed using AtlasViewer software^34^ (Fig. 1). The probe consisted of 16 sources and 24 long-separation detectors (~ 3 cm distance from the source) and 8 short-separation detectors (~ 8 mm distance from the source). This probe geometry resulted in 60 channels in total (52 long-separation and 8 short-separation channels) covering from inferior frontal cortex to the posterior parietal region on both hemispheres.

### Experimental design

**Figure 2:**
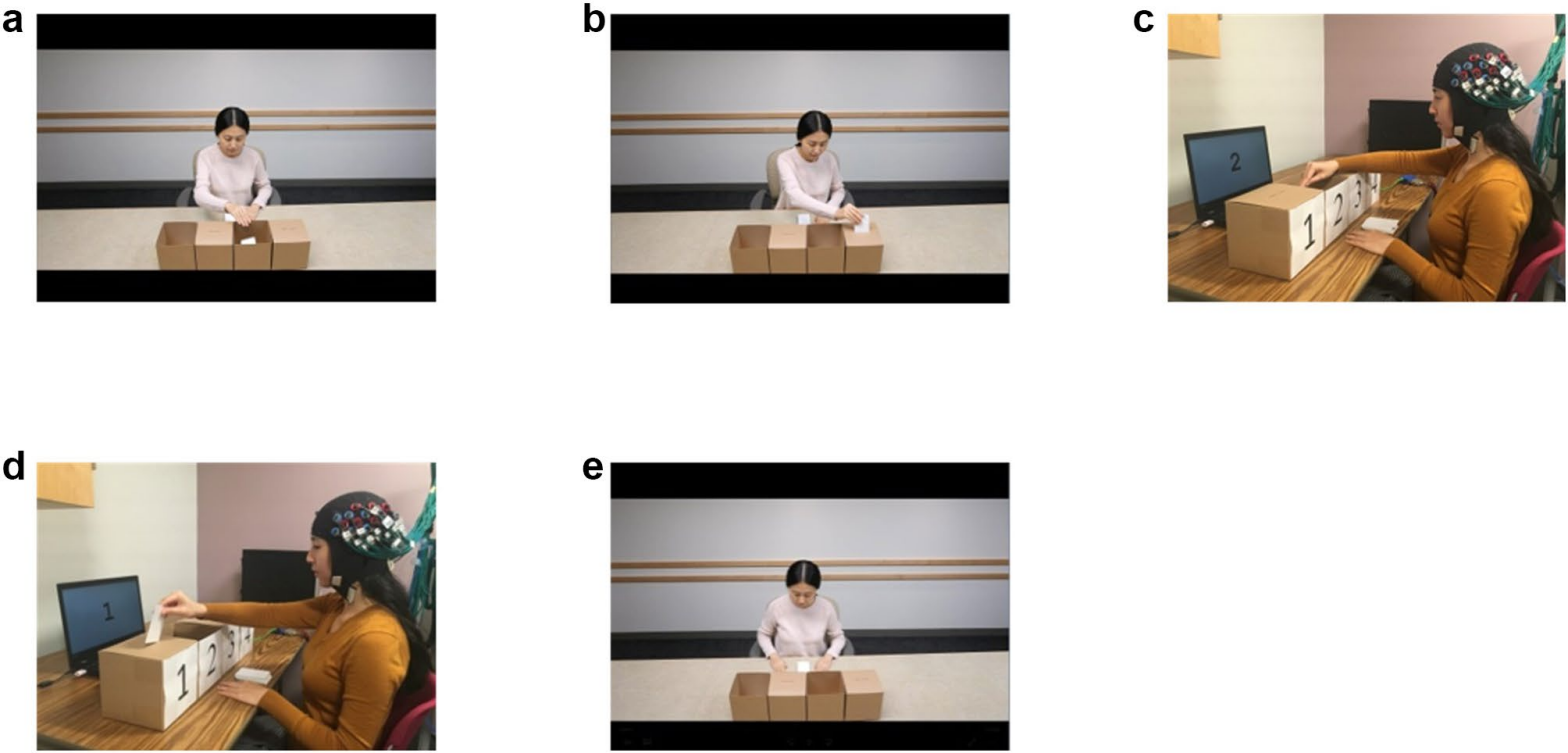
Observation and execution of a hand motor task. (**a**) Observation of the simple condition, (**b**) observation of the complex condition, (**c**) execution of the simple condition, (**d**) execution of the complex condition, (**e**) control condition (observing a video with no hand action). The written informed consent was obtained from the individual for the publication of this image.

Participants performed a task which included the observation and execution of a motor action at two levels of motor complexity. The task consisted of five conditions, three of which involved observing a video clip and two involved the execution of a hand motion. The demonstration of the experimental design is shown in Fig. 2. The written informed consent was obtained from the individual for the online open-access publication of this image. The participants sat in front of a table on which there were four boxes, two open and two with narrow slots, and a computer screen. For the observation conditions, the screen projected video clips, while for the execution conditions, the screen displayed a number to cue participants which box to place the card into. In the video clips used for the observation conditions, an actor was using her right hand to grasp a card and either put it into one of the two open boxes (simple hand action) or insert it into one of the two boxes with narrow slots on the lid (complex hand action) (Fig. 2a,b). In the execution conditions, the participants performed these same hand actions using their right hand (Fig. 2c,d). The fifth control condition involved a video with no hand action (Fig. 2e), thus providing visual input equivalent to the observation conditions.

An event-related design was utilized for this study. To maximize statistical power, we generated three different onset time vectors through optimizing the design matrix for five conditions with an inter-stimulus interval (ISI) of 2–17 s^35^. The study consisted of three runs: each run started with a 15 s baseline followed by 35 trials (7 trials per condition) and each trial lasted 4 s. The entire run lasted 350 s. During the initial 15 s of baseline as well as during the inter-stimulus intervals, the subjects watched a grey screen with a black cross in the middle. Following the NIRS acquisition, the 3D position of all sources and detectors were obtained by a 3D digitizer for each participant (Polhemus Inc., VT, USA). The digitized data of three participants were excluded from further analysis due to interference issues occurred during the digitization process. The inter-subject variability (n = 18) of the 3D locations of all the optodes can be found in the supplementary material (Table S1 and Figure S1).

### Data analysis and statistics

Data analyses were carried out using the open source software HOMER2^36^ implemented in MATLAB 2019a (Mathworks, Natick, MA, USA). The raw NIRS signals were converted into optical density (OD). The channels with optical density lower than 80 dB or higher than 140 dB were excluded from further analysis because for our fNIRS system the signal to noise ratio is typically less than 10 when the signal is below 80 dB, and the signal saturates above 140 dB. Motion artifacts in the optical density data were detected and corrected by a hybrid method based on the spline interpolation method and Savitzky–Golay filtering^37^. The data were then bandpass filtered with a six-order Butterworth filter at 0.01–0.5 Hz in order to remove drifts and high-frequency oscillations. The changes in OD signal were then converted into the concentration changes of oxygenated-hemoglobin (HbO) and deoxygenated-hemoglobin (HbR) by employing the modified Beer–Lambert law with a partial pathlength factor of 6^38–40^.

The hemodynamic response function (HRF) was estimated by a General Linear Model (GLM) approach that uses ordinary least squares (hmrDeconvHRF_DriftSS function in HOMER2). The HRF was modeled as a series of consecutive Gaussian functions with a standard deviation of 1 s and their means were separated by 1 s over the time range of − 2 s to 15 s. In order to eliminate systemic interferences in the calculation of the hemodynamic response, for each long-separation channel, the short-separation channel that has the highest correlation with that long-separation channel was found and this short-separation channel time series is then used as a regressor in the General Linear Model to model the systemic physiology in that long-separation channel^41^. The rationale behind this is that short-separation channels measure signals from superficial layers, while long-separation channels contain information from both superficial layers and the brain. Therefore, by regressing out the short-separation channels, a more robust estimation of the underlying hemodynamic response to brain activation can be achieved. Baseline correction was performed by subtracting the mean of the HRF between − 2 and 0 s from the rest of the HRF.

**Figure 3:**
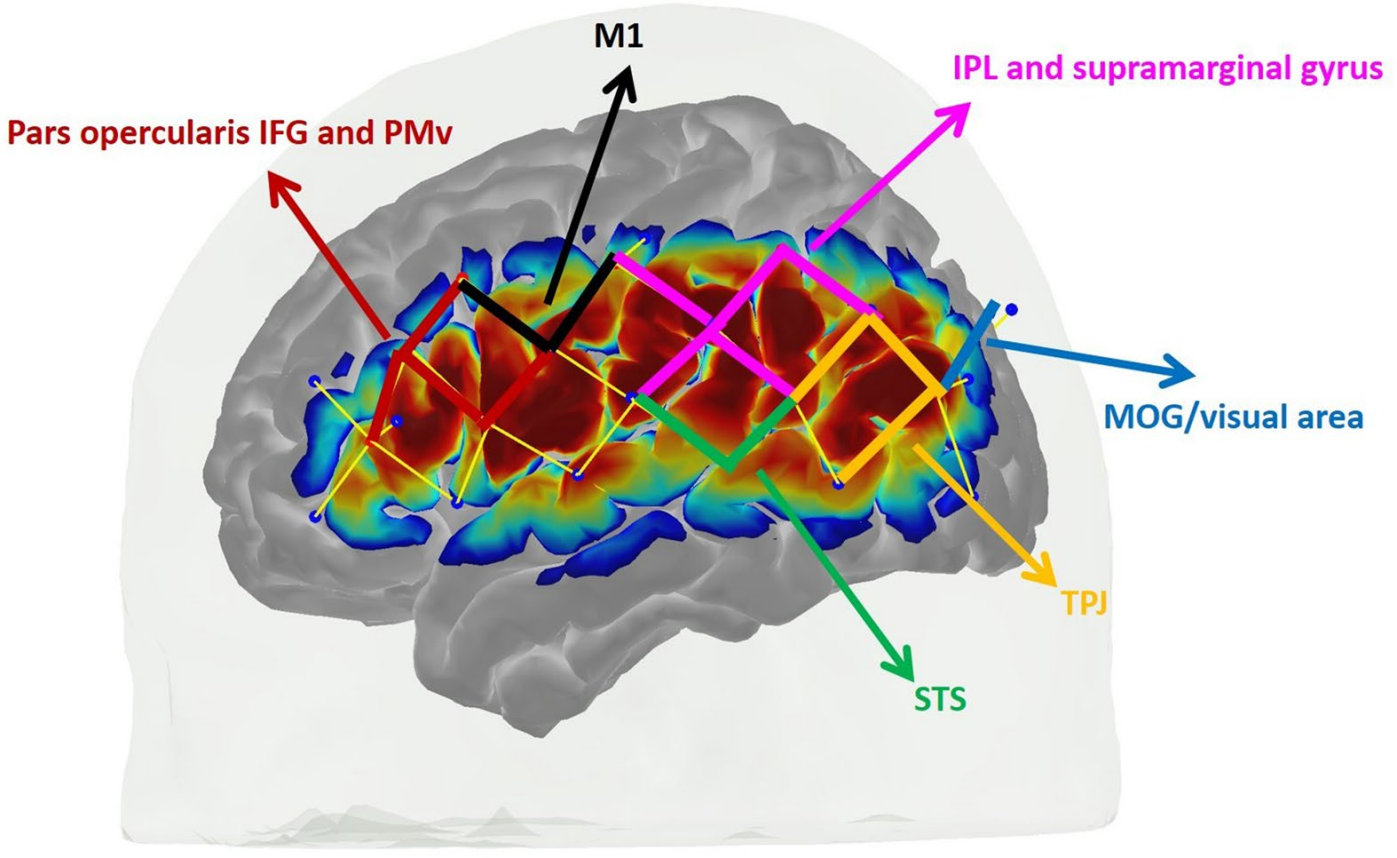
Regions of interest. Six ROIs were identified at each hemisphere: pars opercularis of inferior frontal gyrus (IFG) and ventral premotor cortex (PMv), primary motor cortex (M1), inferior parietal lobule (IPL) and supramarginal gyrus, middle occipital gyrus (MOG), temporo-parietal junction (TPJ) and superior temporal sulcus (STS). Channels for each ROI are shown with a different color.

The 3D locations of five anatomical landmarks (nasion, inion, left and right pre-auricular points and Cz) were used to scale the Colin brain atlas^42^ to each participant’s head size using AtlasViewer^34^. This allowed estimation of the MNI coordinates of optodes and channels by registering the 3D location of optodes to the head surface and projecting them to the cortex. The MNI coordinates of channels generated in AtlasViewer are presented in Table S2. Based on the MNI coordinates of channels, six regions of interest (ROIs) for each hemisphere were identified (Fig. 3; Table S2). Subsequently, the hemodynamic responses from channels within one ROI were averaged and a paired Student’s *t* test was computed at each time point across the whole time course of the hemodynamic response to determine statistically significant differences between the conditions. The channels utilized for each ROI are indicated in Table S2. We carried out our statistical analyses using HbO because it has better signal–noise ratio, however we have also presented HbR changes in our results. We performed paired *t* tests on the mean peak activation of HRF across the conditions. Although the peak activation of HRF occurred in different time ranges across the conditions, the time window used to extract and average the peak activation within the comparison between two conditions were kept the same. Shapiro–Francia normality test has been performed before the paired *t* test. Multiple comparison correction was applied in both statistical comparisons to each hemisphere using the Benjamini–Hochberg method with a false discovery rate of 0.05^43^. Confidence bands have been calculated as follows. The 95% confidence interval was calculated at each time point via multiplying the standard error across subjects by the *t*-statistics at the 95% confidence level using MATLAB (Mathworks, MA, USA). After obtaining the confidence interval point-wise, the confidence band was drawn for the whole-time traces of HbO and HbR.

One can obtain a cortical topography of the brain activity using diffuse optical imaging methods. In order to obtain such cortical maps for HbO and HbR, an atlas head model was registered to the subject’s head via the digitized points of sources and detectors. This allowed us to obtain a more accurate estimation of the location of brain activations. We, then, obtained a sensitivity profile of each channel across the registered atlas head using a GPU-based Monte Carlo photon migration simulations^44^. Sensitive profile (A) is a matrix that transforms from the voxel space of localized changes, X, to the measurement space y.

1 $$y = AX.$$

The X above is then obtained by solving the inverse problem as described previously^45–47^:

2 $$X = A^{T} (AA^{T} + \lambda I)^{ - 1} y.$$

**X** is the unknown HbO and HbR brain maps, ***A*** is the sensitivity matrix of the registered atlas, ***I*** is the identity matrix and y is the mean of the estimated hemodynamic response (HbO/HbR) within a certain time range (the relevant time ranges are provided in the results) at each channel. We set the regularization parameter ***λ*** to 0.01. The HbO and HbR maps were displayed during the baseline (− 2 to 0 s) and during every three seconds after the stimuli onset.

## Results

**Figure 4:**
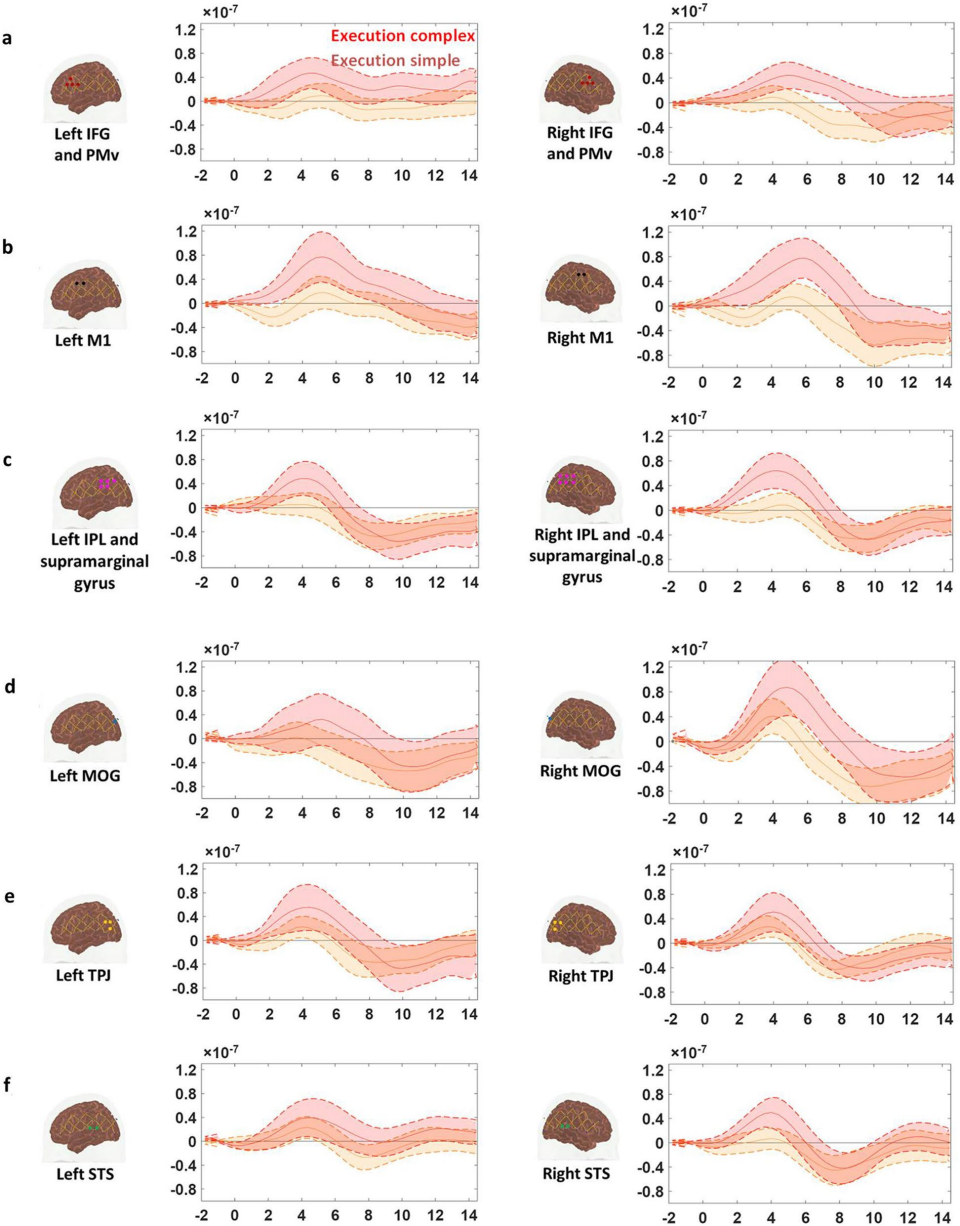
Group average HbO changes (M) for execution simple (orange line) vs. execution complex (red line) hand action. The brain on the upper left of each panel indicates the channel configuration of the ROI presented below. The shaded areas confined by dashed lines show 95% confidence bands.

**Figure 5:**
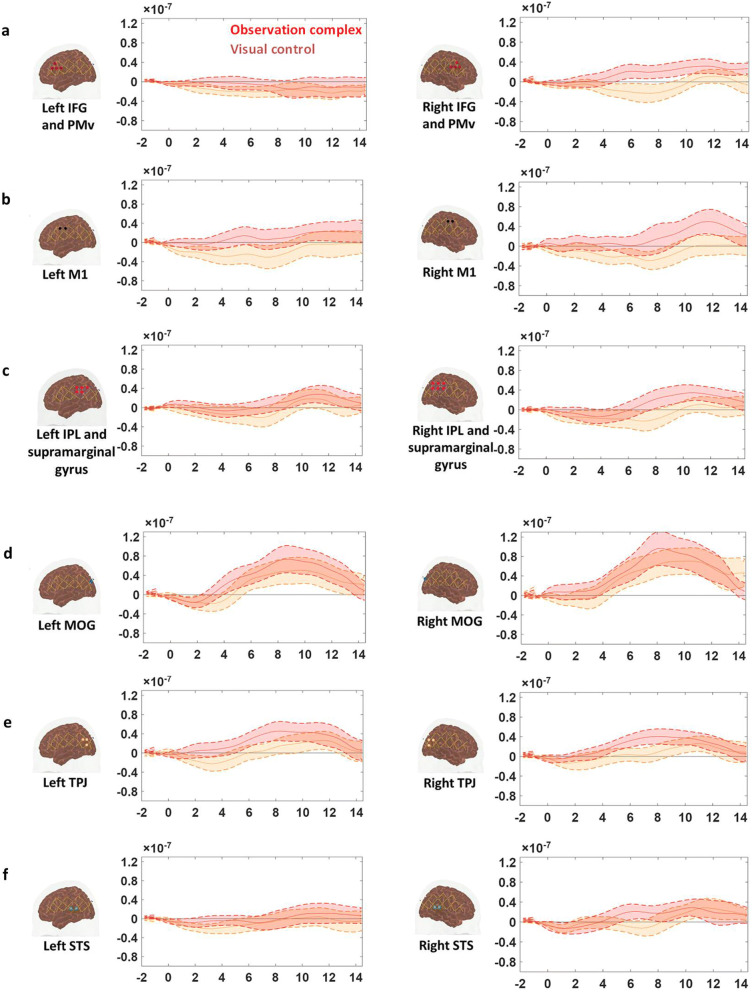
Group average HbO changes (M) for observation of complex (red line) and visual control (orange line) conditions. The brain on the upper left of each panel indicates the channel configuration of the ROI presented. The shaded areas confined by dashed lines show 95% confidence bands.

**Figure 6:**
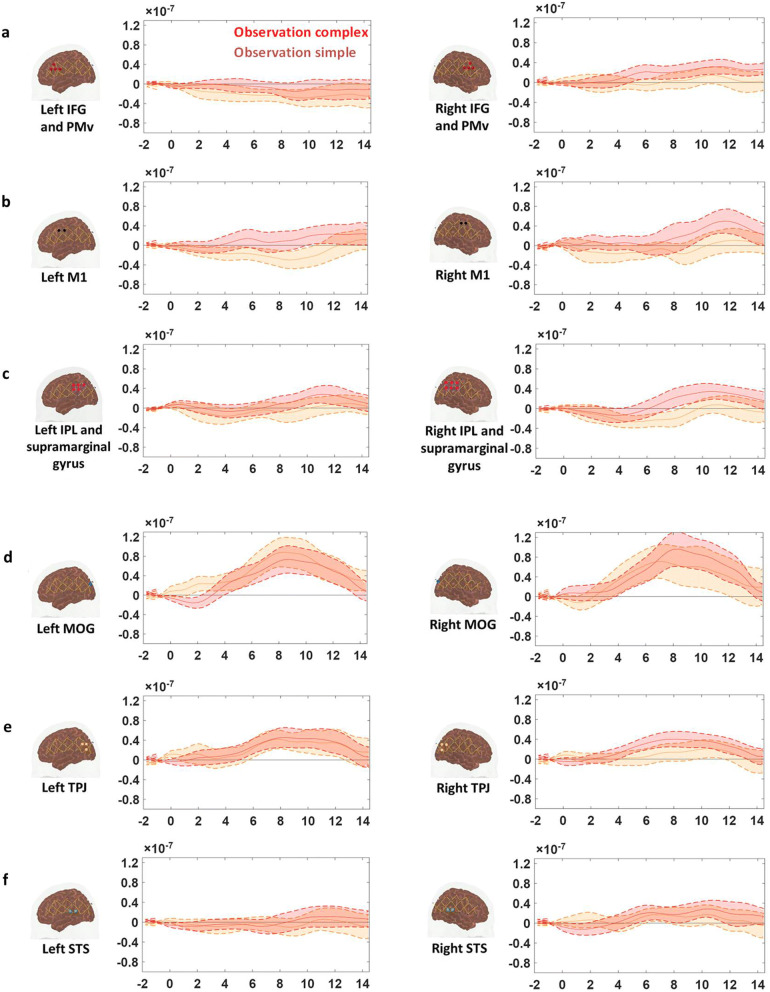
Group average HbO changes (M) for observation simple (orange line) and observation complex (red line). The brain on the upper left of each panel indicates the channel configuration of the ROI presented below. The shaded areas confined by dashed lines show 95% confidence bands.

**Figure 7:**
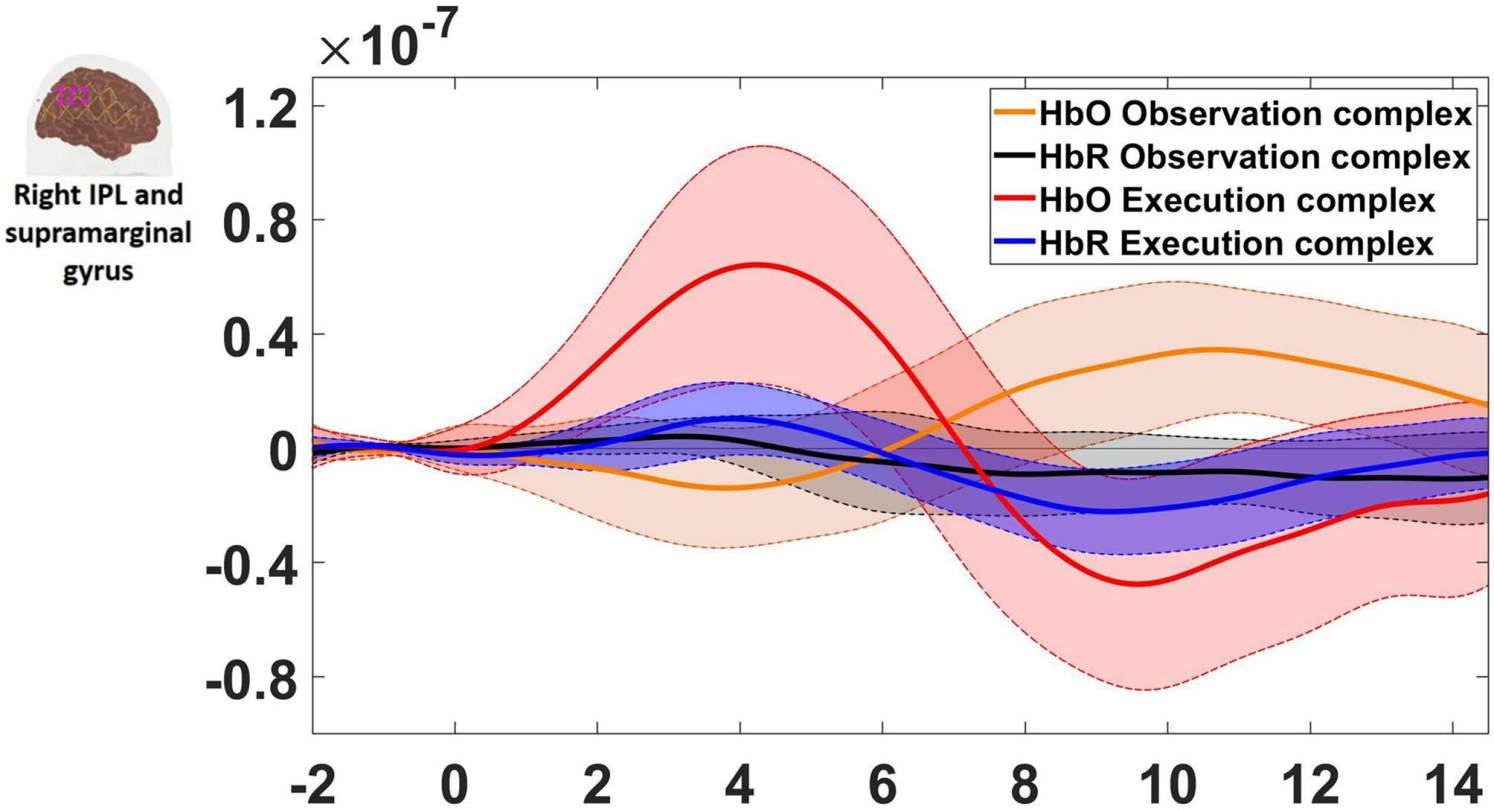
An example of the HRF time course of HbO and HbR changes (M) during the observation complex (orange and black lines respectively) and the execution complex tasks (red and blue lines). The ROI is depicted on the left. The shaded areas confined by dashed lines show 95% confidence bands.

**Figure 8:**
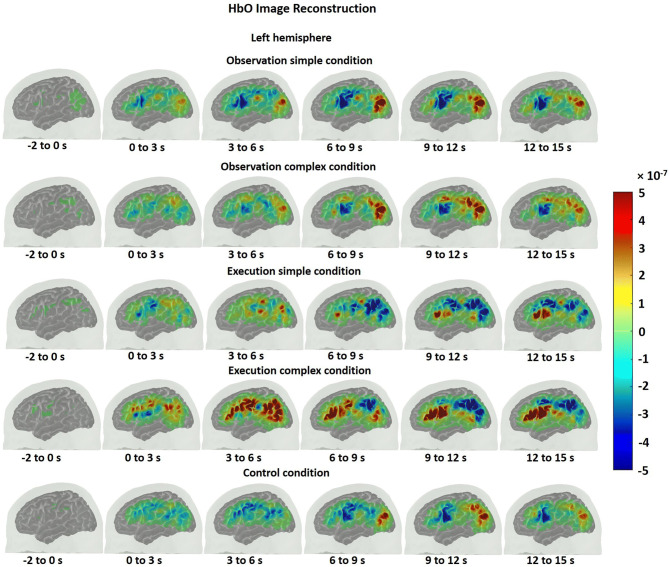
Group mean HbO image reconstruction on the left hemisphere of the brain surface for five conditions. Each reconstructed image is averaged over the time course of the HRF provided below. The color bar is concentration change in Ms.

**Figure 9:**
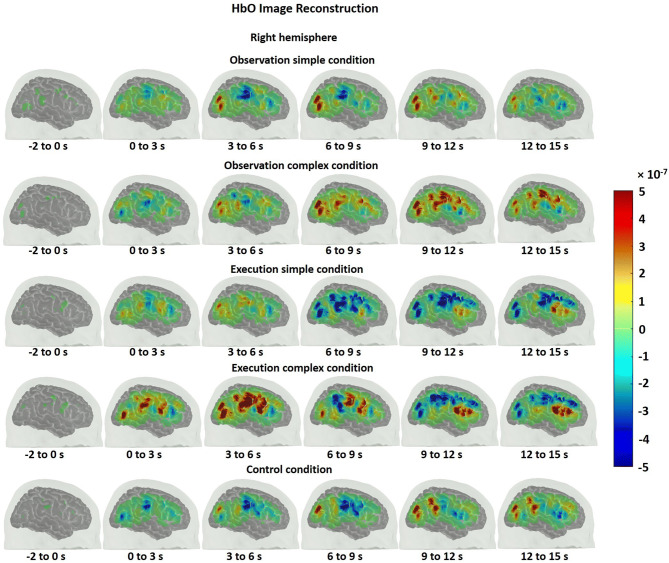
Group mean HbO image reconstruction on the right hemisphere of the brain surface for five conditions. Each reconstructed image is averaged over the time course of the HRF provided below. The color bar is concentration change in Ms.

**Figure 10:**
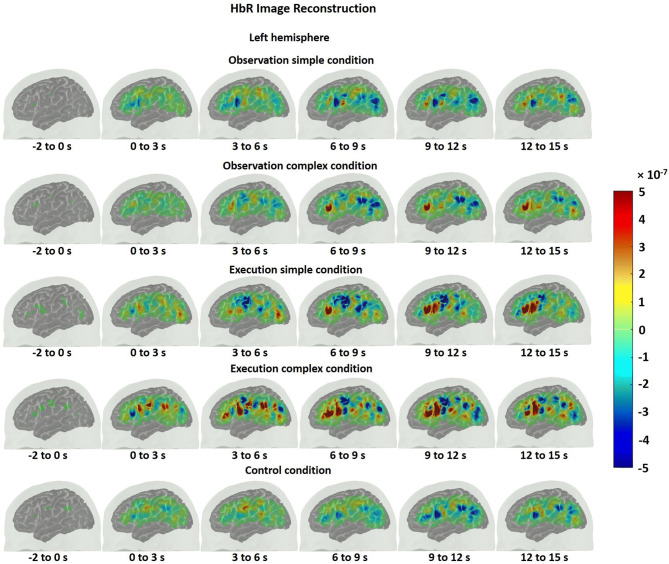
Group mean HbR image reconstruction on the left hemisphere of the brain surface for five conditions. Each reconstructed image is averaged over the time course of the HRF provided below. The color bar is concentration change in Ms.

**Figure 11:**
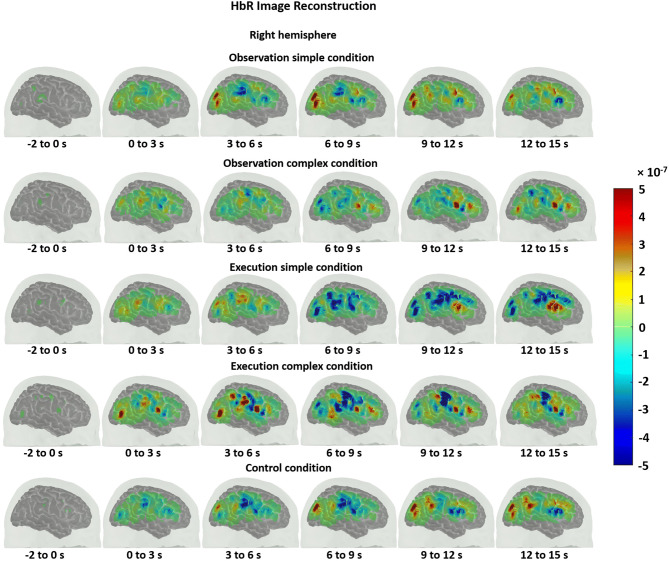
Group mean HbR image reconstruction on the right hemisphere of the brain surface for five conditions. Each reconstructed image is averaged over the time course of the HRF provided below. The color bar is concentration change in Ms.

The HbO time traces with confidence bands within each comparison of two conditions at each ROI are depicted in Figs. 4, 5, 6. The results for the comparisons of the mean peak activation of HbO time courses between two conditions are shown in Table S3. An example of differing temporal characteristics of the HRF with confidence bands between conditions is shown in Fig. 7. Image reconstruction of the group average concentration changes of oxygenated-hemoglobin and deoxygenated-hemoglobin are displayed in Figs. 8, 9, 10, 11 for several succeeding time periods of the hemodynamic response. The channel-wise group average temporal traces of oxy-hemoglobin changes comparing experimental conditions can be found in Figures S2 to S6 in the supplementary material.

### Motor complexity during action execution is more strongly represented in the ipsilateral hemisphere

Peak activation of HbO changes was extracted and averaged from 2–8 s and compared between execution simple and complex conditions. In comparison to the execution of the simple hand action, there was a significant increase in the concentration changes of HbO during the execution of the complex hand action at three ROIs in the right hemisphere: pars opercularis IFG and PMv, IPL and supramarginal gyrus, MOG, (pair *t* test, FDR corrected, p-values < 0.05) (Fig. 4a, c, d, right column; Table S3, fourth column). The one exception to this ipsilateral lateralization was the M1 where the response was strongly bilateral (Fig. 4b). Although the HbO response was higher during the execution complex condition compared to the execution simple condition in the left IPL/supramarginal gyrus as well as right STS, the statistical significance levels did not survive FDR correction.

### Brain response showed lateralization during the observation task

Peak activation of HbO changes was extracted and averaged from 7–10 s and compared between observation complex and visual control conditions. Compared to the control condition, HbO changes were significantly higher during observation of complex hand actions at three ROIs in the right hemisphere: pars opercularis IFG/PMv, M1, IPL/supramarginal gyrus (pair *t* test, FDR corrected, p-values < 0.05) (Fig. 5a–c, right column; Table S3, third column). We did not observe any significant difference between observation of simple hand actions and the control condition in any of the ROIs.

### Differing motor complexity is represented in the IFG/PMv and M1 during observation

The comparison of mean peak activation of HbO changes (averaged from 6 to 12 s) between observation complex and simple conditions showed significant differences (see Table S3, second column) in right pars opercularis IFG/PMv (Fig. 6a, right column) as well as bilateral M1s (Fig. 6b, both left and right columns) (pair *t* test, FDR corrected, p-values < 0.05). The contrast in the right IPL/supramarginal gyrus was also marginally significant after FDR correction (Fig. 6c, right column).

### Motor complexity is represented in visual areas during execution but not during observation

In the middle occipital gyrus, the HbO change during the execution of the complex hand action was significantly higher than the HbO change during the execution of the simple hand action (Fig. 4d, right column) (pair *t* test, FDR corrected, p-value < 0.05). On the other hand, the activation in MOG did not differ among observation and visual control conditions (Figs. 5d and 6d, right column).

### Observation and execution produced HRFs with different temporal characteristics

Examples of HRF time courses of HbO and HbR changes during observation of complex and execution of complex conditions are shown in Fig. 7. While the HbO change peaked around 8 to 12 s during the observation task, it peaked at an earlier time point, around 4 s after stimulus onset, during the execution task, following a “typical” HRF pattern. Another difference in HRFs between conditions was the lag between the HbO peak and the HbR peak. Here, the HbR peak refers to the maximal decrease in the concentration changes of HbR. The HbO peak and HbR peak timings matched better for the observation condition, while the HbR peak was much more delayed compared to the HbO peak during execution. The time to peak was extracted for HbO and HbR changes across subjects and statistically compared between conditions. The HbO changes peaked significantly earlier during the execution task than during the observation task (pair *t* test, p-value < 0.05). The time to peak for HbR changes did not significantly differ between conditions, but the HbO and HbR peak timings did match better for the observation condition (*Mean*_HbOobservation_ = 9.70 ± 2.90 s; *Mean*_HbRobservation_ = 8.22 ± 4.91 s), whereas the HbR peak was delayed around 3 s in contrast to the HbO peak for the execution task (*Mean*_HbOexecution_ = 5.76 ± 3.59 s; *Mean*_HbRexecution_ = 9.06 ± 2.15 s). The same phenomenon is also reflected in Figs. 8, 9, 10, 11 which show the HbO and HbR image reconstruction. In the observation complex condition, the peak activation of HbO occurred in time windows of 6–15 s, and the corresponding HbR peak happened in the same time windows, whereas in the execution of the complex condition, HbO peaked in the time range of 3–9 s, while the HbR peak was around 6–12 s.

## Discussion

In this study, we aimed to investigate the brain correlates of motor complexity during the observation and execution of actions as represented by measuring the brain activity of individuals when they performed observation and execution tasks where goal-directed hand actions differed in motor complexity. Our main findings are that (1) motor complexity during execution as represented by the difference between the brain response to simple and complex hand actions, is more strongly represented in ipsilateral pars opercularis IFG/PMv, IPL/supramarginal gyrus and MOG as well as bilateral M1s, (2) motor complexity during observation is represented primarily in bilateral M1s as well as right pars opercularis IFG/PMv, (3) motor complexity is represented in visual areas during execution but not during observation and (4) HRF temporal characteristics were different for the observation and execution tasks.

The previous literature suggests that increased motor complexity in an action execution task would result in stronger activation of the pars opercularis IFG, PMv and IPL—regions shown to be engaged during planning–preparation period—due to the increased motor preparation and planning involved^48–53^. In order to assure that the two hand actions in this study (simple vs complex) indeed differed in motor complexity, we looked at the brain responses during the execution of these hand actions. Ipsilateral pars opercularis IFG, PMv and IPL as well as bilateral M1s showed significantly higher activation during execution of the complex hand actions compared to the simple hand actions. This first step confirmed that the two execution tasks actually required different levels of motor planning and thus differ in motor complexity.

The differences in brain activity observed during the execution tasks with different complexity were also present during mere observation of tasks with different complexity. Pars opercularis IFG/PMv and IPL, as well as, M1 in the right hemisphere showed significantly higher activation during observation of complex hand actions compared to the control condition which involved a still image. On the other hand, the brain response during simple versus complex hand actions differed primarily in right pars opercularis IFG/PMv and M1s bilaterally. Previous studies have found mixed results regarding the engagement of M1 during the observation of actions. Some electroencephalogram (EEG), magnetoencephalogram (MEG) and transcranial magnetic stimulation (TMS) studies on humans as well as single neuron recordings and quantitative C-2-deoxyglucose (2DG) studies on monkeys indicated the facilitation and activation of M1 during the observation of actions^8, 54–63^. On the contrary, one single neuron recording study on monkeys showed that the discharge of M1 output neurons were modest or suppressed during action observation, along with one fMRI study which showed reduced BOLD signal during hand action observation^64,65^. Although it is still not clear whether the activity of M1 is modulated through the mental rehearsal of the observed actions or the inhibition of the actual execution, all previous work indicates the involvement of M1 during action observation. In this study, the significant difference found in M1 between observation simple and complex conditions additionally provided the evidence for the engagement of M1 during action observation through retrieving the motor representation of varying motor complexity in M1 during the processing of observed hand actions.

Except in the motor cortex, we have observed motor complexity resulted in strong ipsilateral lateralization, as represented by a significant contrast between the brain responses during the execution simple versus complex hand actions as well as observation of complex hand actions versus visual control condition. Aziz-Zadeh and colleagues previously observed that, the activity in IFG and IPL, although relatively bilateral, was indeed stronger in the ipsilateral hemisphere during the imitation of both left and right hand actions^66^. Nevertheless, we did not compare the hemodynamic responses between two hemispheres directly and the ipsilateral activity we observed is related to the contrast of varying motor complexities.

Compared to the execution of the simple task, there was significantly greater activation in the visual area MOG, during the execution of the complex task. On the other hand, although the brain response during simple as well as complex observation conditions was higher than the brain response during the control condition, the difference was not statistically significant. It has been proposed that visual areas are connected to motor areas for the sensory guidance of movement^67^, hence the greater activation in MOG during the execution of the complex task could be explained by more intense processing of spatial and temporal features of visual inputs in this visual area in order to guide the execution of the complex hand actions (e.g. when looking for the narrow slot and inserting the card precisely into it). This result also suggests that the visual inputs during observation and control conditions are equivalent so that differences during observation of simple and complex hand actions or control condition detected in other regions cannot be merely explained by differences in visual processing.

We also found that the temporal characteristics of the HRF differed evidently for observation and execution conditions (see Figs. 7, 8, 9, 10, 11). While the HbO response started to rise gradually at the onset of the action and peaked right after the offset of the action during the execution condition, the HbO change was relatively slow and reached its peak value only several seconds after the “offset” of the action during the observation condition. One possible explanation for the different temporal characteristics of the HRF is the neural mechanism underlying motor preparation. The visual number cues on the boxes that initiated the action execution may have prompted motor preparation immediately in the execution of the complex condition, leading to the relatively rapid increase in HbO in the M1, pars opercularis of IFG/PMv and IPL/supramarginal gyrus. Another possible explanation is that motor imagery could have been involved during the observation conditions. The relatively delayed HbO peak during observation of the complex condition may result from the accumulation of hemodynamic responses that occurred during action observation and motor imagery that may have taken place right after the video presentation (~ 4 s after the onset). This interpretation is supported by some of our study participants’ feedback, who confirmed their attempt in imagining themselves performing the motor action right after the video presentation. Further support for this interpretation comes from previous fMRI and fNIRS studies. Wriessnegger and colleagues observed that compared to motor execution, the brain response to motor imagery peaked approximately 2 s later^68^. Other neuroimaging work also found a similar slower response during motor imagery in precentral gyrus and posterior parietal region^69,70^.

On a related note, compared with the timing of the HbO peak, the timing of the corresponding HbR peak was delayed approximately 3 s for action execution in the complex condition but not for observation in the complex condition (see Figs. 8, 9, 10, 11). A similar HbR delay has also been found in motor cortex during a finger tapping task^71^. The converging evidence here implies that different experimental conditions may result in HRFs with different temporal characteristics and more importantly may considerably diverge from a canonical HRF shape. These two points about HRF time course are critical in the sense that most of the fMRI studies as well as some fNIRS studies model the hemodynamic response using a canonical HRF. Using such a fixed model may bias the results considering how different the brain response may look from region to region as well as between different experimental tasks. Thus, we recommend to look at HRF time courses before making such assumptions.

Our study has several limitations given its current design. First, although all participants were asked to use their right hands during the execution tasks, not all the participants were right-handed. Thus, we also performed all the analysis excluding the two left-handed subjects. We did not find any change in the results presented in this work. Second, our study did not record behavioral data. Although this is a limitation, since all participants were able to perform both actions correctly without making any mistakes, i.e. the performance during execution was at ceiling, our results were still interpretable.

## Conclusions

We have observed increased activity in pars opercularis IFG/PMv and M1 during the observation of complex versus simple hand actions as well as in pars opercularis IFG and PMv, IPL and supramarginal gyrus, M1 and visual areas during the execution of a complex motor task in contrast to a simple motor task. Our findings suggest that the processing of motor complexity involves not only M1, but also the areas IFG, PMv and IPL. These brain regions together comprise the brain correlates of motor complexity during action execution and perception, implicating one of the brain mechanisms underlying action understanding and motor cognition.

## Supplementary information

Supplementary information

Supplementary information 2

Supplementary information 3

## Data Availability

The datasets generated during and/or analyzed during the current study are available from the corresponding author on reasonable request.
