# Supplementary material for: Brain correlates of motor complexity during observed and executed actions
Source: Sci Rep. 2020 Jul 3;10:10965. doi: 10.1038/s41598-020-67327-5 (PMC7335074; doi:10.1038/s41598-020-67327-5)
Supplement: Supplementary file 3 — Supplementary information 3 [file 41598_2020_67327_MOESM3_ESM.pdf]

Brain Correlates of Motor Complexity during Observed and Executed Actions

Xinge Li[1,2], Manon A. Krol[3*], Sahar Jahani[4,5*], David A. Boas[2,4], Helen Tager-Flusberg[3] and Meryem A. Yücel[2,4,**]

[1] *School of Psychology, South China Normal University, Guangzhou, China*
[2] *Neurophotonics Center, Department of Biomedical Engineering, Boston University, Boston, MA, USA*
[3] *Center for Autism Research Excellence, Department of Psychological and Brain Sciences, Boston University, Boston, MA, USA*
[4] *MGH/HST Athinoula A. Martinos Center for Biomedical Imaging, Department of Radiology, Massachusetts General Hospital, Harvard Medical School, Charlestown, MA, USA*
[5] *Control and Intelligent Processing Center of Excellence, School of Electrical and Computer Engineering, College of Engineering, University of Tehran, Tehran, Iran*
*** They contributed equally**
**** Correspondence:**
Meryem A. Yücel
mayucel@bu.edu

Supplementary materials

Table S1. Mean and standard deviation of the 3D position of optodes (short-distance detectors not included) across subjects (n=18).

Optodes	X	Y	Z	Standard Deviation (mm)
1	62.64	125.33	63.03	13.70
2	48.45	122.39	93.21	10.95
3	45.61	121.56	124.34	9.03
4	47.25	123.55	151.90	9.48
5	55.27	129.72	180.03	10.33
6	59.81	90.96	93.29	11.86
7	57.78	86.61	125.45	11.04
8	60.47	92.55	156.01	11.27
9	207.32	133.97	178.53	8.38
10	212.09	130.5	153.07	8.43
11	214.49	126.04	121.70	10.88
12	208.87	126.27	87.83	13.87
13	191.21	125.57	59.01	18.03
14	202.27	99.56	158.75	9.88
15	207.91	97.05	125.61	10.38
16	201.19	94.55	90.57	13.62
17	68.13	144.41	52.01	15.02
18	52.14	139.44	80.15	11.32
19	42.55	139.41	107.41	9.91
20	44.96	141.15	138.20	9.57
21	47.64	146.69	163.16	9.00
22	58.83	148.30	191.03	9.87
23	82.29	111.39	49.16	15.69
24	59.31	108.33	76.31	12.11

25	49.23	104.20	110.35	10.22
26	50.98	103.77	140.28	9.77
27	54.61	111.34	168.23	10.29
28	68.10	117.61	194.67	10.92
29	201.94	150.24	191.32	8.63
30	212.35	147.25	159.98	8.60
31	214.44	141.61	134.07	9.57
32	214.77	139.62	102.02	11.92
33	203.40	143.67	78.05	26.41
34	185.71	146.67	53.34	34.10
35	195.52	121.27	191.65	8.94
36	205.26	114.32	169.69	9.04
37	211.37	114.57	139.17	9.81
38	211.89	110.25	105.20	12.19
39	196.70	108.01	72.67	15.68
40	173.28	109.89	49.28	19.78

Table S2. MNI coordinates and brain regions of channels.

Channels	MNI	Label name	Brodmann area	ROIs
1	44 -67 13	Temporal_Mid_R	Right BA 19	
2	63 -64 18	Temporal_Mid_R	Right BA 39	Right TPJ
3	44 -62 21	Temporal_Mid_R	Right BA 39	
4	51 -76 28	Occipital_Mid_R	Right BA 39	Right MOG
5	39 -50 26	Angular_R	Right BA 39	Right TPJ
6	68 -51 19	Temporal_Mid_R	Right BA 39	
7	56 -41 17	Temporal_Sup_R	Right BA 22	Right STS
8	60 -50 29	SupraMarginal_R	Right BA 39	Right TPJ
9	48 -42 26	SupraMarginal_R	Right BA 40	Right IPL and supramarginal gyrus
10	64 -23 18	SupraMarginal_R	Right BA 40	Right STS
11	73 -12 16	Postcentral_R	Right PrimSensory (1)	
12	62 -15 24	SupraMarginal_R	Right BA 40	
13	52 -21 30	SupraMarginal_R	Right PrimSensory (1)	Right IPL and supramarginal gyrus
14	56 -11 30	SupraMarginal_R	Right PrimMotor (4)	
15	70 2 15	Postcentral_R	Right PrimMotor (4)	
16	57 10 13	Frontal_Inf_Oper_R	Right BA 44	
17	60 1 30	Postcentral_R	Right BA 6	Right pars opercularis IFG and PMv
18	64 13 28	Precentral_R	Right BA 6	Right pars opercularis IFG and PMv
19	63 21 12	Frontal_Inf_Oper_R	Right BA 44	
20	63 40 12	Frontal_Inf_Tri_R	Right BA 46	
21	48 22 17	Frontal_Inf_Oper_R	Right BA 44	
22	58 22 26	Frontal_Inf_Tri_R	Right BA 44	Right pars opercularis IFG and PMv
23	62 39 24	Frontal_Inf_Tri_R	Right BA 9	

24	58 -50 43	Angular_R	Right BA 39	Right IPL and supramarginal gyrus
25	45 -35 41	SupraMarginal_R	Right BA 40	Right IPL and supramarginal gyrus
26	61 -24 47	SupraMarginal_R	Right BA 40	Right IPL and supramarginal gyrus
27	59 -12 45	Postcentral_R	Right PrimMotor (4)	Right M1
28	45 -17 44	Postcentral_R	Right PrimMotor (4)	
29	53 0 40	Precentral_R	Right BA 6	Right M1
30	55 13 36	Precentral_R	Right BA 6	Right pars opercularis IFG and PMv
31	-30 30 13	Frontal_Inf_Tri_L	Left BA 45	
32	-56 19 12	Frontal_Inf_Oper_L	Left BA 45	
33	-35 16 22	Frontal_Inf_Oper_L	Left BA 44	
34	-53 36 20	Frontal_Inf_Tri_L	Left BA 46	
35	-47 21 24	Frontal_Inf_Tri_L	Left BA 44	Left pars opercularis IFG and PMv
36	-41 6 13	Rolandic_Oper_L	Left BA 44	
37	-56 -1 10	Rolandic_Oper_L	Left BA 6	
38	-52 10 24	Frontal_Inf_Oper_L	Left BA 44	Left pars opercularis IFG and PMv
39	-61 4 25	Precentral_L	Left BA 6	Left pars opercularis IFG and PMv
40	-63 -16 14	Temporal_Sup_L	Left PrimSensory (1)	
41	-41 -24 12	Temporal_Sup_L	Left PrimAuditory (41)	Left STS
42	-49 -20 18	Rolandic_Oper_L	Left PrimSensory (1)	

43	-46 -17 24	Postcentral_L	Left PrimSensory (1)	
44	-51 -25 27	SupraMarginal_L	Left BA 40	Left IPL and supramarginal gyrus
45	-47 -43 14	Temporal_Mid_L	Left BA 39	Left STS
46	-40 -45 14	Temporal_Mid_L	Left BA 39	
47	-43 -44 25	SupraMarginal_L	Left BA 39	Left IPL and supramarginal gyrus
48	-56 -55 29	SupraMarginal_L	Left BA 39	Left TPJ
49	-37 -58 14	Temporal_Mid_L	Left BA 39	Left TPJ
50	-37 -66 11	Occipital_Mid_L	Left BA 19	
51	-42 -71 23	Occipital_Mid_L	Left BA 19	
52	-40 -61 25	Angular_L	Left BA 39	Left TPJ
53	-31 -66 28	Occipital_Mid_L	Left BA 39	Left MOG
54	-40 11 28	Frontal_Inf_Oper_L	Left BA 8	Left pars opercularis IFG and PMv
55	-38 3 28	Precentral_L	Left BA 6	Left M1
56	-38 -16 36	Postcentral_L	Left PrimMotor (4)	Left M1
57	-44 -23 37	SupraMarginal_L	Left PrimSensory (1)	Left IPL and supramarginal gyrus
58	-47 -23 42	Parietal_Inf_L	Left PrimSensory (1)	
59	-41 -40 36	SupraMarginal_L	Left BA 40	Left IPL and supramarginal gyrus
60	-51 -54 39	Parietal_Inf_L	Left BA 39	Left IPL and supramarginal gyrus

Note: Brodmann area for each MNI coordinate were acquired from http://sprout022.sprout.yale.edu/mni2tal/mni2tal.html.

Table S3. Paired t-test on the averaged peak activation of HbO changes between conditions (p-values after FDR correction).

ROIs	Observation simple VS Observation complex	Observation complex VS Visual control	Execution simple VS Execution complex
	Averaged from 6-12 sec	Averaged from 7-10 sec	Averaged from 2-8 sec
Left IFG and PMv	0.40	0.61	0.08
Right IFG and PMv	0.01	<.01	<.01
Left M1	0.01	0.20	0.04
Right M1	0.01	0.05	<.01
Left IPL and supramarginal gyrus	0.40	0.20	0.08
Right IPL and supramarginal gyrus	≈0.05	0.05	0.01
Left MOG	0.54	0.20	0.12
Right MOG	0.42	0.20	0.04
Left TPJ	0.82	0.09	0.08
Right TPJ	0.31	0.18	0.15
Left STS	0.66	0.75	0.20

Right STS	0.66	0.18	0.07

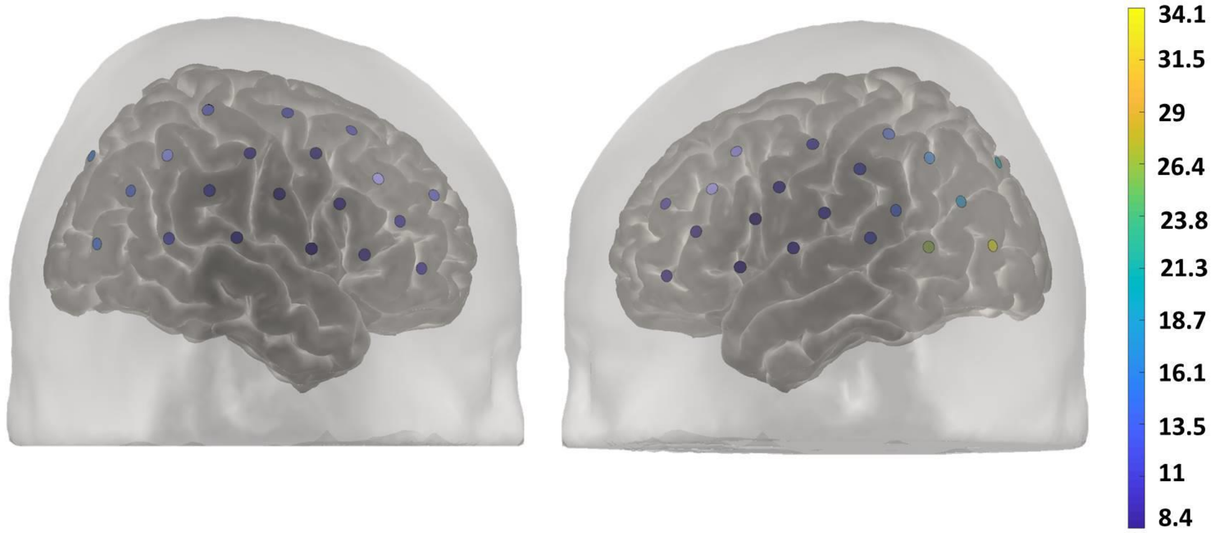

Fig S1. Inter-subject variability of the 3D position of all the optodes (short-distance detectors not included). Each dot represents an optode. The standard deviation of the 3D location of optodes across subjects is indicated by the color of the dot (in mm).

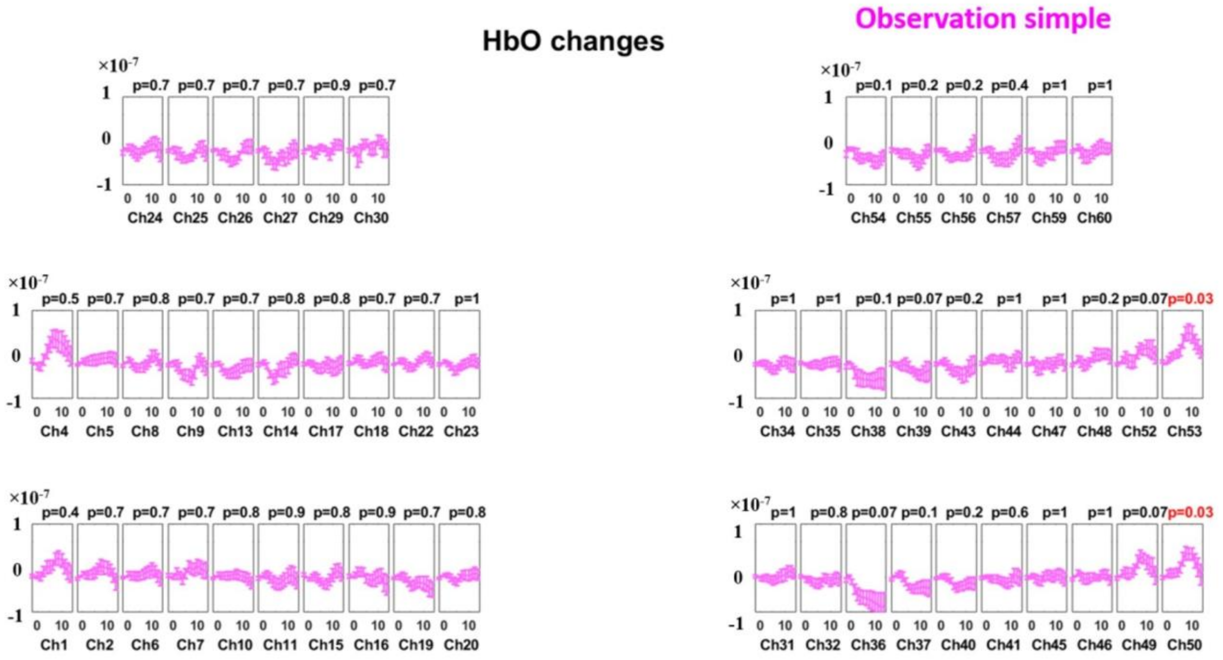

Fig S1. *Paired-t* test between observation simple condition and baseline (FDR corrected). The time range for averaging peak activation of observation simple condition is from 7s to 11s, while the time range of baseline is from -2s to 0s. Channel 53 in the ROI of left MOG and Channel 50 had significant activation. Channel 4 in right MOG and channel 52 in left TPJ had significant activation before FDR correction.

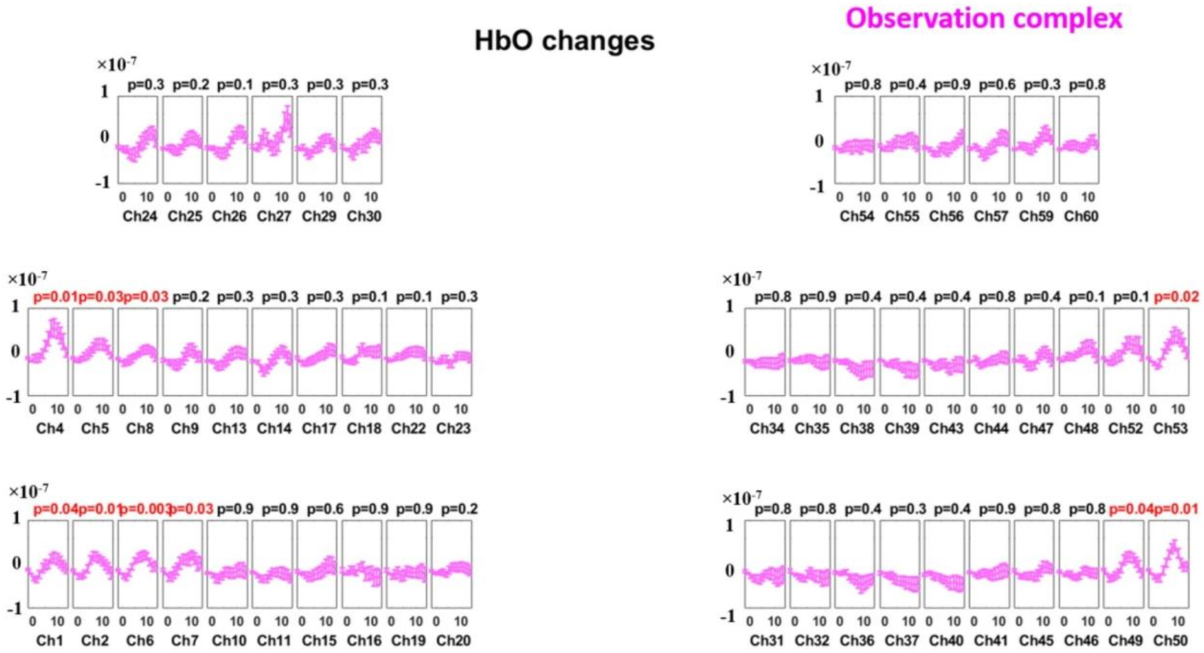

Fig S2. *Paired-t* test between observation complex condition and baseline (FDR corrected). The time range for averaging peak activation of observation complex condition is from 7s to 11s, while the time range of baseline is from -2s to 0s. Some significant channels were located in the ROIs of bilateral MOG and TPJ and right STG. Channel 18 in right IFG and PMv and channel 48 and 52 in left TPJ had significant activation before FDR correction.

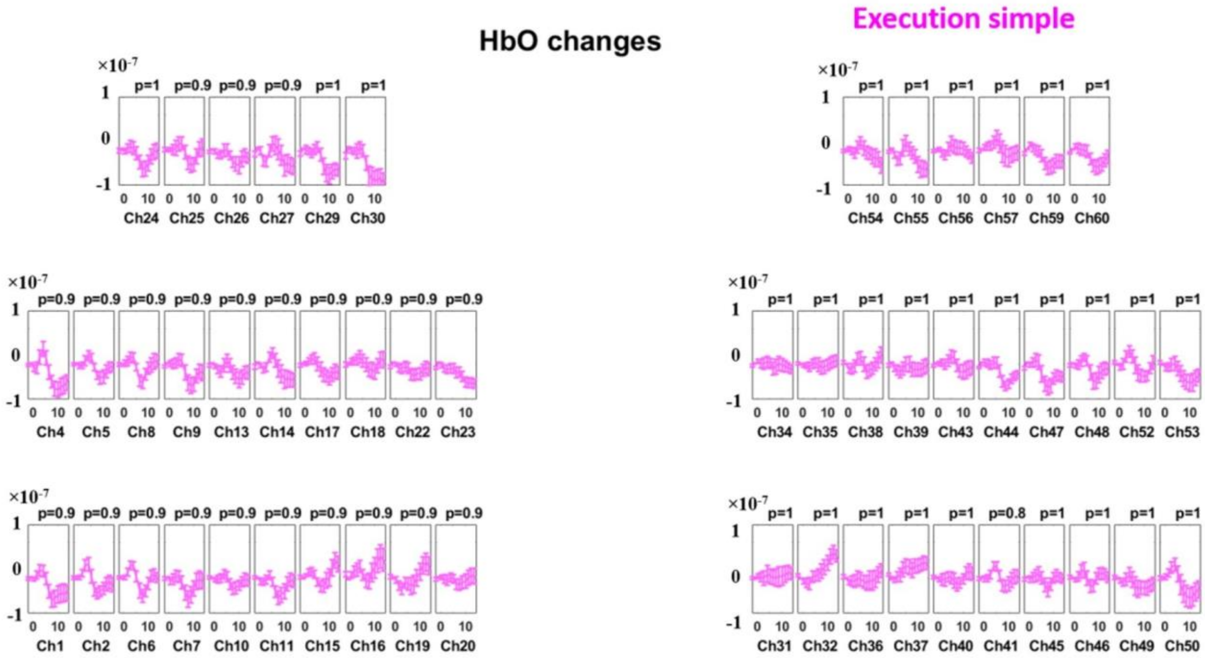

Fig S3. *Paired-t* test between execution simple condition and baseline (FDR corrected). The time range for averaging peak activation of execution simple condition is from 2s to 6s, while the time range of baseline is from -2s to 0s. No significant channel was found.

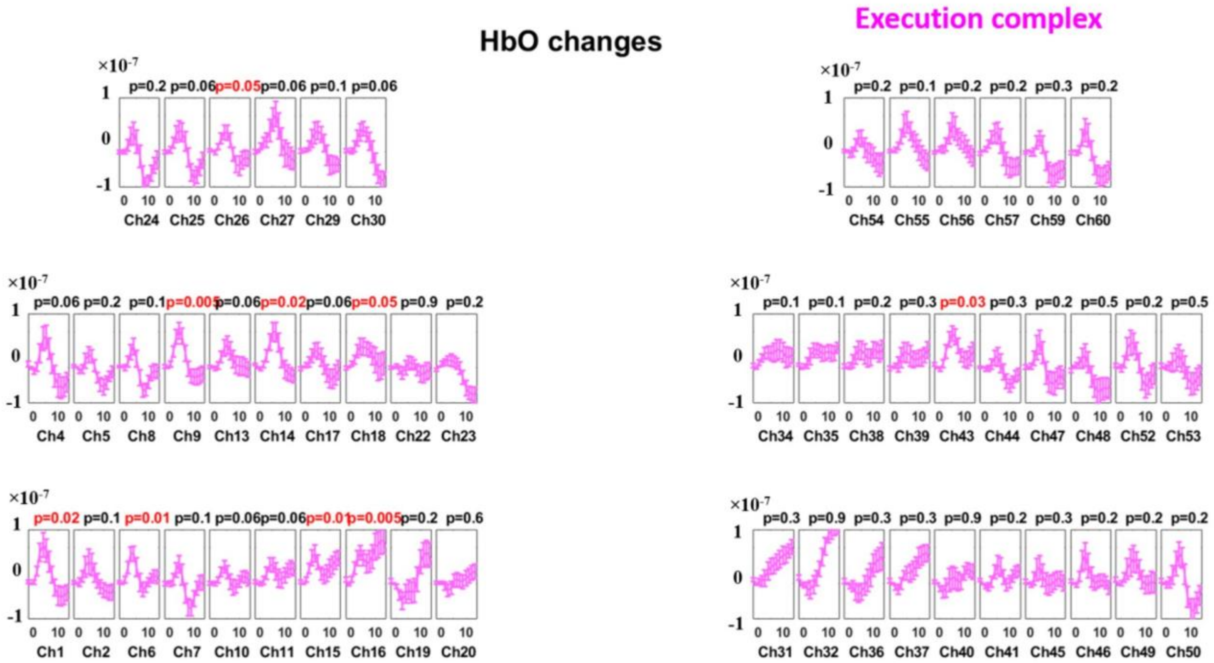

Fig S4. *Paired-t* test between execution complex condition and baseline (FDR corrected). The time range for averaging peak activation of execution complex condition is from 2s to 6s, while the time range of baseline is from -2s to 0s. Some significant channels were located in the ROIs of right pars opercularis IFG and PMv along with right IPL and supramarginal gyrus. Channel 35 in left pars opercularis IFG and PMv, channel 27 and 55 at bilateral M1s, channel 13, 25 and 26 in right IPL and supramarginal gyrus, channel 10 in right STS had significant activation before FDR correction.

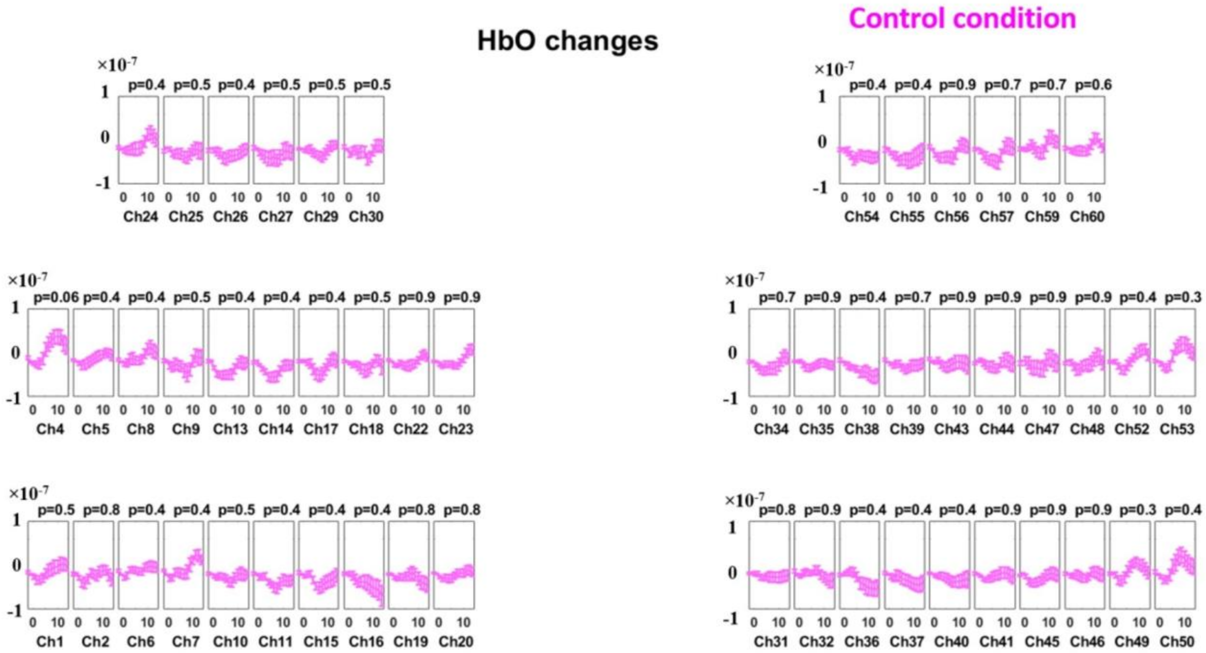

Fig S5. *Paired-t* test between control condition and baseline (FDR corrected). The time range for averaging peak activation of observation simple condition is from 7s to 11s, while the time range of baseline is from -2s to 0s. No significant channel was found.
